# City of London Hospital for Diseases of the Chest

**Published:** 1892-06-04

**Authors:** 


### FESTIVAL DINNERS AND HOSPITAL MEETINGS.

### CITY OF LONDON HOSPITAL FOR DISEASES OF THE CHEST.

The festival dinner in aid of the above hospital was held at the Hotel Metropole on the 24th ult., the Earl of Strafford, Lord Lieutenant of the County of Middlesex, presiding. Among those present were over one hundred representatives of the several societies who contribute to the funds of the hospital.

The Chairman, in proposing the toast, "Prosperity to the City of London Hospital for Diseases of the Chest," said that twenty-five years ago he had had the honour of presiding at a previous festival dinner, and he hoped they would believe him when he said that the sympathy with that institution which he felt in the year 1865 was felt equally by him in the year 1892. That institution was first established in the month of June, 1848, by an out-patients' branch being founded in Liverpool Road. In June, 1851, the foundation-stone of the hospital in Victoria Park was laid by the late lamented Prince Consort, and the hospital was opened for the reception of patients in the year 1855. New wings had been subsequently added in 1863 and 1871. During 1891 1,236 in-patients had been treated, making the total number of this class of patients who had been admitted since 1855 25,000. Those resident in the hospital during 1891 averaged 122 daily. During the same period 15,529 out-patients had been treated, making the number treated since the foundation of the institution in 1848 489,890. The average attendance of out-patients each week was 1,066. The annual expenditure was £10,000, towards which there were annual subscriptions (including the grants from the Hospital Sunday and Saturday Funds) to the tune of £3,500, and invested funds of only £300. The past year's expenditure had exceeded the income by £2,848. Contracts, as he had no doubt his readers were aware, had been entered into, and were now in course of completion, for the erection of new blocks for improving the ventilation and sanitary arrangements. A special feature of the institution well worthy of note by him was the fact that it was so liberally and generously supported by the working men of the neighbourhood, who gave £900 a year towards the income of the hospital. Possibly no hospital did so much for the sick and suffering as the Chest Hospital. No less than one-ninth of the mortality of all ages, and one-fifth of that of adults of the United Kingdom was attributed to consumption, and when they looked back on the last three years and thought of the frightful suffering which had been inflicted by that terrible disease, the influenza, he was quite certain that everyone must feel that a great hospital like the one whose cause he was pleading was doing an incalculable amount of good. (Applause.) In conclusion, he exhorted his hearers to try and enlist the sympathy and support of their relatives and friends in the great and good work which, under the blessing of Providence, that institution had managed to do. An institution, he said, which, since 1848, had contributed to the relief of half-a-million of people would not, he was sure, be allowed to fail for want of funds. (Applause.)

A total contribution of £2,584 was announced by the Secretary.

